# Short-term exposure to ambient air pollution and hospital visits for IgE-mediated allergy: A time-stratified case-crossover study in southern China from 2012 to 2019

**DOI:** 10.1016/j.eclinm.2021.100949

**Published:** 2021-06-10

**Authors:** Xiangqing Hou, Huimin Huang, Haisheng Hu, Dandan Wang, Baoqing Sun, Xiaohua Douglas Zhang

**Affiliations:** a Faculty of Health Sciences, University of Macau, Macao, China; b Department of Allergy and Clinical Immunology, State Key Laboratory of Respiratory Disease, National Clinical Research Center of Respiratory Disease, Guangzhou Institute of Respiratory Health, First Affiliated Hospital of Guangzhou Medical University, Guangzhou Medical University, Guangdong, China

**Keywords:** Air pollutions, Atopic diseases, IgE-mediated allergy, Short-term exposure, Subgroup analysis

## Abstract

**Background:**

Because of the limited epidemiological evidence on the association between acute air pollutants and allergy, there is a need to investigate this association, especially between the short-term exposure to air pollution and the serum Immunoglobulin E (IgE)-mediated allergy.

**Methods:**

A total of 39,569 IgE test results and demographic characteristics were obtained in the First Affiliated Hospital of Guangzhou Medical University between August 2012 and September 2019. Ninety-nine specific allergens were tested according to clinical diagnosis. The logistic regression was used to assess the effects of CO, NO_2_ and PM_2.5_ exposure on the risk of sensitization to specific inhalant/food allergens. Generalized additive models with multivariate adjustments were utilized to model the exposure-response relationship. Stratified analyses were performed to estimate the reliability of correlations in various subgroups.

**Findings:**

Single-pollutant models indicate that the 3-day moving average (lag_2–4_) of CO, PM_2.5_ or NO_2_ is associated with the increased risk for allergic diseases related to specific inhaled allergens. In multi-pollutant models, the adjusted Odds Ratio (OR) 95% (Confidence Interval, CI) increases by 8% (95% CI, 2%–15%) for per increment of 0.2 mg/m^3^ in CO levels, and rises by 8% (95% CI, 2%–13%) for each increase of 16.3 μg/m^3^ in PM_2.5_ concentration. The associations are stronger in youngsters (<18, years) but not significantly different by gender. Particularly, a significantly stronger association between PM_2.5_ exposure and hospital visits for inhaled allergy is observed in patients who are exposed to lower concentration of SO_2_ (<10.333 μg/m^3^) and higher levels of NO_2_ (≥42.0 μg/m^3^), as well as patients enrolled after 2017.

**Interpretation:**

The short-term exposure to CO/PM_2.5_ increases the number of hospital visits for IgE-mediated allergy, especially for the sensitization to specific inhalant allergens. Therefore, to prevent inhaled allergies, the public policy for controlling air pollution needs to be considered seriously.

**Funding:**

This study was supported by the University of Macau (grant numbers: FHS-CRDA-029–002–2017 and MYRG2018–00,071-FHS) as well as the Science and Technology Development Fund, Macau SAR (File no. 0004/2019/AFJ and 0011/2019/AKP). This work was also supported by the National Natural Science Foundation of China (81,871,736), the National Key Technology R&D Program (2018YFC1311902), the Guangdong Science and Technology Foundation (2019B030316028), the Guangzhou Municipal Health Foundation (20191A011073), and the Guangzhou Science and Technology Foundation (201,804,020,043).

**Research in context**

**Evidence before this study**

Although there is suggestive evidence supporting the association between the development of Immunoglobulin E (IgE) sensitization and long-term or early-life exposure to air pollution, few studies investigate the associations between the short-term exposure to ambient air pollutants and the prevalence of sensitization to specific allergic sources, especially in the large city-specific level in China.

**Added value of this study**

To our knowledge, this is the first epidemiology study to comprehensively investigate the associations between the daily hospital visits for IgE-mediated allergy and the short-term exposure to air pollution in southern China. We discover that the 3-day moving average (lag_2–4_) of CO/PM_2.5_ is associated with the increased risk for allergic diseases related to specific inhalant allergens. The associations are stronger in youngsters (<18, years). Particularly, a significantly stronger association between PM_2.5_ exposure and hospital visits for inhaled allergy is observed in the patients who are exposed to lower concentration of SO_2_ (<10.333 μg/m^3^) and higher levels of NO_2_ (≥42.0 μg/m^3^), as well as patients enrolled after 2017.

**Implications of all the available evidence**

The present study contributes to the currently limited epidemiologic evidence regarding the associations between the short-term exposure to air pollution and the daily hospital visits for IgE-mediated allergy. Our findings should enable public health policymakers to recommend safety limitations on the exposure levels of outdoor air pollutants for those susceptible to inhaled allergies at the city-specific level which is similar to Guangzhou.

Alt-text: Unlabelled box

## 1 Introduction

Allergic diseases have become common chronic diseases in both children and adults, and it poses tremendous economic and health burdens among general populations [1,2]. A national cross-sectional study performed in ten Chinese provinces reports that the prevalence of asthma is up to 4.2% [3]. In addition, recent literature [4] shows that the prevalence of allergic rhinitis (AR) is continuously rising and has become a leading cause of allergic diseases in China. A survey [5] conducted in southern China displays that the concomitant prevalence of asthma and AR has reached 5.33%. Atopic Dermatitis (AD) can occur in people of almost all ages, which increases the psychosocial burden of the patients and the risk of mental health disorders [6]. Despite the significant burden of these diseases, no universally effective strategy can prevent and control the occurrence of allergic diseases to date. However, early identification and limiting specific exposure factors that can increase the risk of sensitization to specific allergens are likely effective to reduce the prevalence of atopic diseases [7,8]. Many epidemiological studies demonstrate that short-term exposure to air pollution is associated with increased numbers of hospital visits for asthma, AR and AD [9,10]. Moreover, several experimental studies [11], [12], [13] suggest that ambient air pollutants are pertinent to the hypersensitivity mediated by mast cells and basophils. Nevertheless, the associations between short-term exposure to outdoor air pollution and daily hospital visits for IgE-mediated allergy are still uncertain because of limited epidemiological evidence.

China, the largest low- and middle-income country, still have much higher air pollution levels though a visible improvement in air quality can be observed recently [14]. It has been suggested that China is the most vulnerable to health and economic burden associated with exposure to ambient air pollution according to Global Disease Estimation in 2017 [15]. Guangzhou, the largest national central city in southern China, has some complicated panels of allergen sensitization patterns [16] as well as continual changes of environmental pollutants [17]. Despite emerging evidence shows that the prevalence of allergic diseases is strongly linked with ambient air pollution, to our knowledge, no study was conducted at the city-specific level in China to comprehensively explore the associations between air pollutants and the risk of daily hospital visits for IgE-mediated allergy. To date, there have been only a few cohort studies targeting the association of atopic diseases, allergic sensitization, and traffic-related air pollution in high-income countries. It has been suggested that the increased risk of sensitization to pollen was associated with PM_2.5_ but not with NO_2_ exposure [18]. Unlike these findings, some specific inhaled allergic molecules, such as birch pollen-specific molecules and timothy grass allergen as well as cat dander-specific molecules, were observed to be closely related to the PM_2.5_/NO_2_ exposure in Europe [19]. These inconsistent results are probably attributable to different levels of air pollution [20], inherent challenges in study design [21], and complicated allergen sensitization patterns in different places [22]. Although suggestive evidence indicates that long-term exposure to air pollution would contribute to the development of allergic sensitization, the effects of short-term exposure to air pollution on the risk of hospital visits for IgE-mediated allergy remain unclear, especially from the city-specific level in China.

In this time-stratified case-crossover study, we comprehensively and quantitatively examine whether transient air pollutants exposure is associated with increased numbers of daily hospital visits for sensitization to specific allergens in Guangzhou, as well as to explore any potential modifying components.

## 2 Materials and methods

### 2.1 Study design

We conduct a time-stratified case-crossover study to investigate the association between short-term air pollution and hospital visits for IgE-mediated allergy*.* This design is widely recognized in studying acute outcomes resulting from short-term exposures, particularly common in studies that focus on ambient pollutants. Given the air pollution on consecutive days is highly correlated, to reduce the selection bias, the case period and the control periods are chosen from the same calendar month and have to be on the same day in different weeks [23]. In this study, on the day of admission for patients who are allergic to one or more common inhaled/food allergens is defined as the risk period (case period), while the control periods are set as 3–4 control days in the same calendar month and the same day of the week with each case day [23]. Since both the case and controls happen in the same month, there are four control periods maximum in each stratum for comprehensive comparisons.

**Fig. 1:**
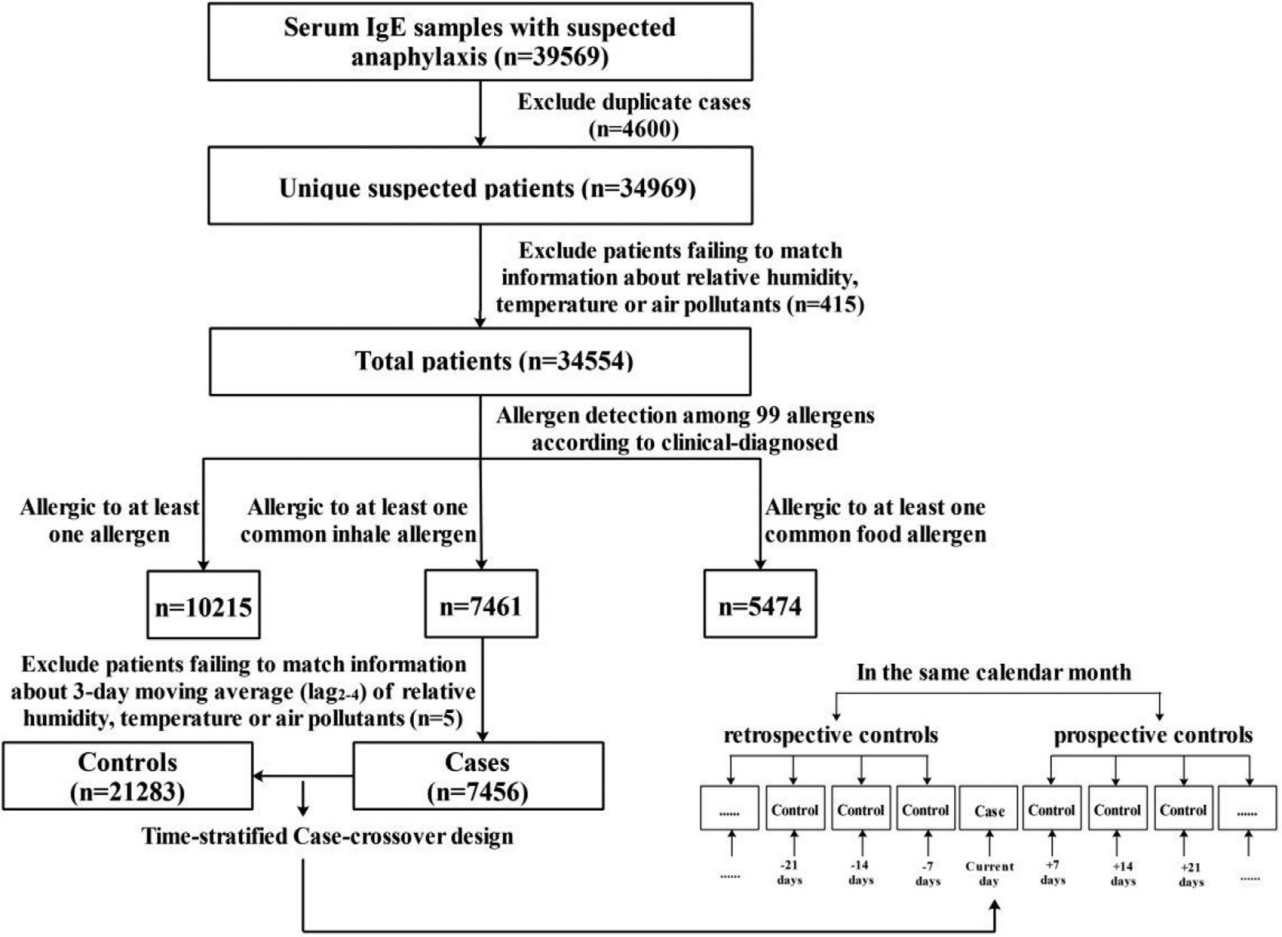
The flowchart of study design. Note: The common inhaled allergens including d1, d2, e1, e5, w6, m3 as well as the common food allergens including f1, f2, f4, f13, f24, f14, f23. Since the case and several controls happen in the same month, there are at most 4 control periods in each stratum.

The flowchart of the study design is shown in Fig. 1. It is of note that a total of 28 days misses information on air pollutants resulting from malfunctioning of air monitoring. Since the missing rate of this study is less than 10%, we exclude those days. Finally, some cases probably match with less than three controls. Additionally, the information in both the case and control groups was collected from the same individuals, therefore, some potential confounders such as age, gender, lifestyle, genetic factors, etc. can be corrected. Choosing multiple controls in the same calendar year, month, and day of the week with case day would be beneficial to reduce the seasonality and time trends of exposure estimates.

### 2.2 Settings

Southern China, with a subtropical monsoon climate (103°E-123°E, 22°N-34°N), which is one of the desirable areas to explore the association between air pollution and the risk of hospital visits for IgE-mediated allergy. It has some complicated panels of allergen sensitization patterns [16] as well as continual changes of environmental pollutants [17]. Guangzhou is an international transportation hub in southern China with more than 15.3 million permanent residents in 2020 and the climate is characterized by warm and rainy spring, a sweltering summer, and dry and moderate winter.

### 2.3 Data collection

We obtained 39,569 IgE test results from all patients with suspected anaphylaxis in the First Affiliated Hospital of Guangzhou Medical University between October 2012 and September 2019. The inclusion criteria were: (1) patients who admitted for sudden allergic reaction, such as asthma, rhinitis, eczema, etc. and then diagnosed as possible allergic diseases by a well-trained physician; (2) completed IgE test according to clinical diagnosis; (3) had been followed-up at least once; (4) visited the hospital between October 2012 and September 2019. Samples with the following situations were excluded: (1) for recurrence patients who repeatedly performed IgE tests within a short time or followed up several times in a period, we just keep the first follow-up record and excluded other duplicate observations; and (2) patients failing to match information about relative humidity, temperature or air pollutants. Based on these criteria, we totally excluded 5020 samples in this study (Fig. 1).

Data on demographics and clinical characteristics of participants, such as age and gender, were extracted from their medical records originating from a structured questionnaire specifically designed for patient's admission. Besides, IgE test was carried out for all serum samples upon diagnosis using ImmuoCAP (Thermo Fisher Scientific Inc., California, USA). Data on allergic sensitization contain indications covering 99 allergens, including some common inhaled allergens (house dust mite, dermatophagoides farinae, aspergillus fumigatus, cat dander, dog dander, mugwort) and food allergens (egg white, milk, wheat, peanut, shrimp, soy, crab). Serum IgE values higher than 0.35 kU/L were defined as positive samples. The diagnostic flowchart of allergic diseases can be seen in Supplement Figure 1, the positive serum IgE samples were classified as IgE-mediated allergy [24]. All allergic information was stored in the serum bank of Allergy Information Repository (AIR-SKLRD) located in the First Affiliated Hospital of Guangzhou Medical University, strictly following the clinical operation guidelines. The detailed information about AIR-SKLRD has been reported in our previous study [25].

### 2.4 Air pollution and meteorological data

We obtained 24 h average concentrations of carbon monoxide (CO), nitrogen dioxide (NO_2_), particulate matter less than 2.5 μm and 10 μm in aerodynamic diameter (PM_2.5_, PM_10_), sulfur dioxide (SO_2_), and the daily maximum of 1 h average ozone concentration (O_3__1 h) in Guangzhou city from Guangdong Provincial Department of Ecology and Environment (http://gdee.gd.gov.cn/). The meteorological data utilized in this study were extracted from China Meteorological Data Sharing Service System (https://data.cma.cn/en). The research area was limited to Guangzhou city and the study period lasted from October 2012 to September 2019. A total of 28 days in this study period are missing with information on air pollutants due to air monitoring malfunction.

### 2.5 Statistical analysis

Sensitivity analysis was utilized to compare the characteristics between included and excluded participants. Median (1^st^ quartile, 3^rd^ quartile) was used to describe the age and total IgE (tIgE) of the participants because of the skewed distribution. Frequency (proportion) was used to report the distribution of gender and age groups (stratified by age of 18 years), and group differences were compared using Chi-squared test. Spearman's correlation analysis was performed to calculate the association between air pollutants and meteorological data, the variable pairs with correlation coefficients greater than or equal to 0.60 were considered to be closely related [26]. The lasso regression model was additionally performed to evaluate the variables selection approach of the Spearman's correlation analysis. Based on the results of previous studies [9,19], 3 common air pollutants (CO, PM_2.5_, NO_2_) and 3 IgE sensitization patterns (allergic to at least one allergen, allergic to at least one of the 6 inhaled allergens, allergic to at least one of the 7 food allergens) were chosen for investigating the associations between air pollution and daily hospital visits for IgE-mediated allergy. In this study, we only comprehensively analyzed the effects of 3 common air pollutants (CO, PM_2.5_ and NO_2_) because the potential biological mechanism between the short-term exposure to those air pollutants and the risk of allergies has been well established. Moreover, the obtained data of air pollutants indicated that PM_2.5_, CO and NO_2_ were the most common air pollutants in Guangzhou. Nevertheless, we still considered the confounding effects of O_3__1 h, SO_2_, etc. on the multi-pollutant models.

In this matched case-crossover study, a series of conditional logistic regression analyses with a single predictor were conducted to estimate the crude effects of CO, PM_2.5_ and NO_2_ exposure on the risk of IgE-mediated allergy in various lag days. The odds ratios (ORs) of various lag days identified that the 3-days moving average (lag_2–4_) of air pollutant concentration has the largest effect on inhaled allergies. To further examine the expose-effect response relationship between air pollution levels (lag_2–4_) and the risk of IgE-mediated allergy, a restrictive cubic spline curve was fitted after being adjusted for relative humidity (with 3 degrees of freedom), mean temperature (with 3 degrees of freedom) and public holidays [27]. The knots of the main continuous exposure were defined according to the percentiles (5^th^ percentile, Median, 95^th^ percentile) of its distribution. The form of conditional logistic regression in a generalized additive model is listed as follows:

(1) $$Y_{t}∼Bernoulli\left(\mu_{t}\right)$$

(2) $$\mu_{t}=e^{\alpha +\beta X_{t}+f\left(\gamma_{t}\right)+f\left(\tau_{t}\right)+\lambda S_{t}+\nu H_{t}}$$

where $Y_{t}$=the number of daily positive IgE test samples on day *t*; $\mu_{t}$= the expected positive IgE samples on day *t; β*= the coefficient for air pollutant concentration; *λ* = the coefficient for stratum; *υ* = the coefficient for public holidays; $\gamma_{t}$ is the average temperature at the lag days 2 to 4; $\tau_{t}$ is the relative humidity at the lag days 2 to 4; *f* is the natural cubic spline function with a degree of freedom 3.

Additionally, the exposure to CO/PM_2.5_ (lag_2-4_) on the risk of inhaled allergies was quantitatively assessed through per standard deviation (SD) and quartile increment after being adjusted for some air pollutants as well as meteorological data. To avoid multicollinearity, in multi-pollutant models, we only included the confounders of Spearman's correlation coefficients less than 0.6 together with main continuous exposure. As for others which were closely related to CO/PM_2.5_ but still had hidden confounding effects, stratified analysis was applied to examine various subgroup differences, and the cut-point median values of CO, PM_2.5_, NO_2_, PM_10_ and SO_2_ were 0.822 mg/ m^3^, 28.677 μg/m^3^, 42.0 μg/m^3^, 47.333 μg/m^3^ and 10.333 μg/m^3^ respectively. Whether the time was in public holidays was recognized as a dichotomous variable, and the research period was stratified into pre- and post- 2017 according to the time distribution of the daily number of hospital visits for acute allergic symptoms (**Supplement Figure 2**). Besides, the differences in gender and age groups (cut-off was 18 years) were also compared in subgroup analysis. Z-test was implemented for assessing the statistical differences between subgroups (e.g., Age<18 *vs*. Age≥18 years). The formula is as follows*.*

(3) $$z=\frac{\beta_{1}-\;\beta_{2}}{\sqrt{S{E_{1}}^{2}+S{E_{2}}^{2}}}$$

where *β_1_* and *β_2_* are the estimates of the effects for the two categories (e.g., Age<18 *vs*. Age≥18 years) and SE_1_ and SE_2_ are their respective corresponding standard errors.

All statistical analyses and figures were performed by SAS 9.4 (Copyright 2002–2012 by SAS Institute Inc., Cary, NC, US). *p* < 0.05 in two-sided test was regarded as significant.

### 2.6 Ethical approval

The research protocol was approved by the First Affiliated Hospital of Guangzhou Medical University ethics committee (Reference number: GYFYY-2016–73).

### 2.7 Patients consent statement

All participants or their parents provided oral and written informed consent for participating in this study, and in conformity with the ethics approval by the First Affiliated Hospital of Guangzhou Medical University ethics committee.

### 2.8 Role of funding

The funders had no roles in study design, data collection, data analysis, interpretation and writing of the report.

## 3 Results

A total of 34,554 suspected allergic participants who met the inclusion criteria were analyzed in this study. The detailed flowchart can be found in Fig. 1. The baseline characteristics of the study samples can be seen in Table 1, which shows that the median of total IgE (tIgE) in patients who were allergic to specific inhalant allergens is much larger than patients to specific food allergens. The median (1^st^ quartile, 3^rd^ quartile) age of all participants is 21.0 (4.0, 51.0) years. It can also be observed that the positive detection rate of females is higher than that of males in all 3 IgE sensitization patterns (*p* < 0.001). Besides, youngsters (<18, years) are probably more vulnerable to IgE-mediated allergy than adults (*p* < 0.001), since the positive detection rates of allergy in youngsters are much larger than that in adults, regardless of specific inhaled or food allergens. The numbers of participants who are allergic to d1, d2, e1, e5, w6, m3, f1, f2, f4, f13, f24, f14 and f23 are 6866, 3018, 253, 504, 166, 628, 2443, 3435, 597, 146, 1549, 122, and 488 respectively.

**Table 1:** Descriptive characteristics of participants.

Variables	Total *N* = 34,554	All allergens£*N* = 10,215	Inhalant allergens¶*N* = 7461	Food allergens§*N* = 5474
tIgE, kU/L	92.44(31.28,265.11)	225.00(92.44,539.00)	294.42(127.54,670.36)	207.22(92.44,515.00)
Gender,%				
Man	14,670	3728(25.4)	2764(18.8)	1872(12.8)
Woman	19,884	6487(32.6)	4697(23.6)	3602(18.1)
*p* valueε	——	<0.001	<0.001	<0.001
Age, year				
<18	16,821	7646(45.5)	5209(31.0)	4583(27.2)
≥18	17,733	2569(14.5)	2252(12.7)	891(5.0)
*p* valueε	——	<0.001	<0.001	<0.001

£ Note: £: Allergic to at least one allergen.

¶ Allergic to at least one common inhalant allergen (d1, d2, e1, e5, w6, m3).

§ Allergic to at least one common food allergen (f1, f2, f4, f13, f24, f14, f23); tIgE: total IgE.

ε The *p*-value is the comparison of detection rates in different allergens patterns between various sex and age groups.

For the study period in Guangzhou, the median (1^st^ quartile, 3^rd^ quartile) of averaged 24 h air pollutant concentration are as follows: 0.8 (0.7, 1.0) mg/m^3^ for CO, 28.0 (20.0, 43.0) μg/m^3^ for PM_2.5_, 48.0 (36.0, 68.0) μg/m^3^ for PM_10_, 10.0 (8.0, 14.0) μg/m^3^ for SO_2_, 42.0 (33.0, 53.0) μg/m^3^ for NO_2_. Besides, the median (1^st^ quartile, 3^rd^ quartile) of the daily maximum O_3__1 h is 46.0 (29.0, 65.0) μg/m^3^. The daily medians (1^st^ quartile, 3^rd^ quartile) of ambient temperature and relative humidity are 24.5 (19.0, 29.0) °C and 75% (66%, 81%), separately. Spearman's correlation analysis between air pollution and weather condition is shown in Table 2. The correlations of CO concentrations with PM_2.5_, PM_10_ and NO_2_ are high. Furthermore, SO_2_, PM_10_ and NO_2_ have a close relationship with PM_2.5_ (*r_s_* > 0.6, *p* < 0.001). In multi-pollutant models, to avoid multicollinearity among correlated air pollutants, Spearman's correlation analysis was utilized to calculate the association between air pollutants and meteorological data. Variable pairs with correlation coefficients greater than or equal to 0.60 were considered to be closely related, and the corresponding confounders were excluded in the final models (Table 3). Moreover, the results of lasso regression also reveal that the final model consists of CO, PM_2.5_ and other 4 confounders (relative humidity, mean temperature, SO_2_ and O_3__1 h), which is consistent with the results of Spearman's correlation analysis (Supplement Figure 3).

**Table 2:** Spearman's correlation coefficients between air pollution and weather conditions in Guangzhou, 2012–2019.

	CO	PM_2.5_	NO_2_	PM_10_	SO_2_	O_3__1h	Relative humidity	Mean temperature
CO	1.000							
PM_2.5_	0.680*	1.000						
NO_2_	0.646*	0.753*	1.000					
PM_10_	0.616*	0.974*	0.755*	1.000				
SO_2_	0.422*	0.653*	0.490*	0.673*	1.000			
O_3__1h	−0.197*	0.286*	−0.001	0.314*	0.299*	1.000		
Relative humidity	−0.034*	−0.436*	−0.175*	−0.450*	−0.395*	−0.551*	1.000	
Mean temperature	−0.563*	−0.398*	−0.468*	−0.325*	−0.084*	0.383*	0.148*	1.000

Note: *p < 0.001.

**Table 3:** Risk of inhaled allergies of participants associated with the 3-day moving average (lag_2–4_) of CO and PM_2.5_ concentrations.

Variables	N 28,739	Cases/Controls 7456/21,283	*Crude*		*Adjusted*	
			*ORs (95% CI)*	*p value*	*ORs (95% CI)*	*p value*
CO concentrations						
Per SD increment of CO (0.2 mg/m^3^)	—	—	1.07(1.02,1.13)	0.007	1.08(1.02,1.15)	0.009¶
Quartile						
Q_1_(0.452~)	7198	1762/5436	1.00(1.00,1.00)	Ref.	1.00(1.00,1.00)	Ref.
Q_2_(0.722~)	7167	1749/5418	0.99(0.90,1.09)	0.792	0.99(0.89,1.09)	0.793¶
Q_3_(0.822~)	7186	1869/5317	1.07(0.96,1.19)	0.243	1.07(0.95,1.20)	0.298¶
Q_4_(≥0.935)	7188	2076/5112	1.18(1.04,1.33)	0.009	1.20(1.04,1.38)	0.012¶
P for trend				0.004		0.006¶
PM_2.5_ concentrations						
Per SD increment of PM_2.5_ (16.3 μg/m^3^)	—	—	1.07(1.03,1.12)	0.001	1.08(1.02,1.13)	0.006£
Quartile						
Q_1_(8.333~)	6958	1729/5229	1.00(1.00,1.00)	Ref.	1.00(1.00,1.00)	Ref.
Q_2_(21.333~)	7612	1861/5751	1.00(0.92,1.10)	0.962	1.00(0.91,1.10)	0.956£
Q_3_(28.667~)	7029	1808/5221	1.06(0.96,1.17)	0.280	1.05(0.94,1.17)	0.385£
Q_4_(≥40.667)	7140	2058/5082	1.15(1.03,1.29)	0.012	1.15(1.00,1.31)	0.048£
P for trend				0.008		0.041£

¶ Note: Adjusted relative humidity, mean temperature, SO_2_ and O_3__1 h;.

£ Adjusted relative humidity, mean temperature and O_3__1 h; ORs: Odds Ratios; Ref.: Reference group; SD: Standard deviation.

**Fig. 2:**
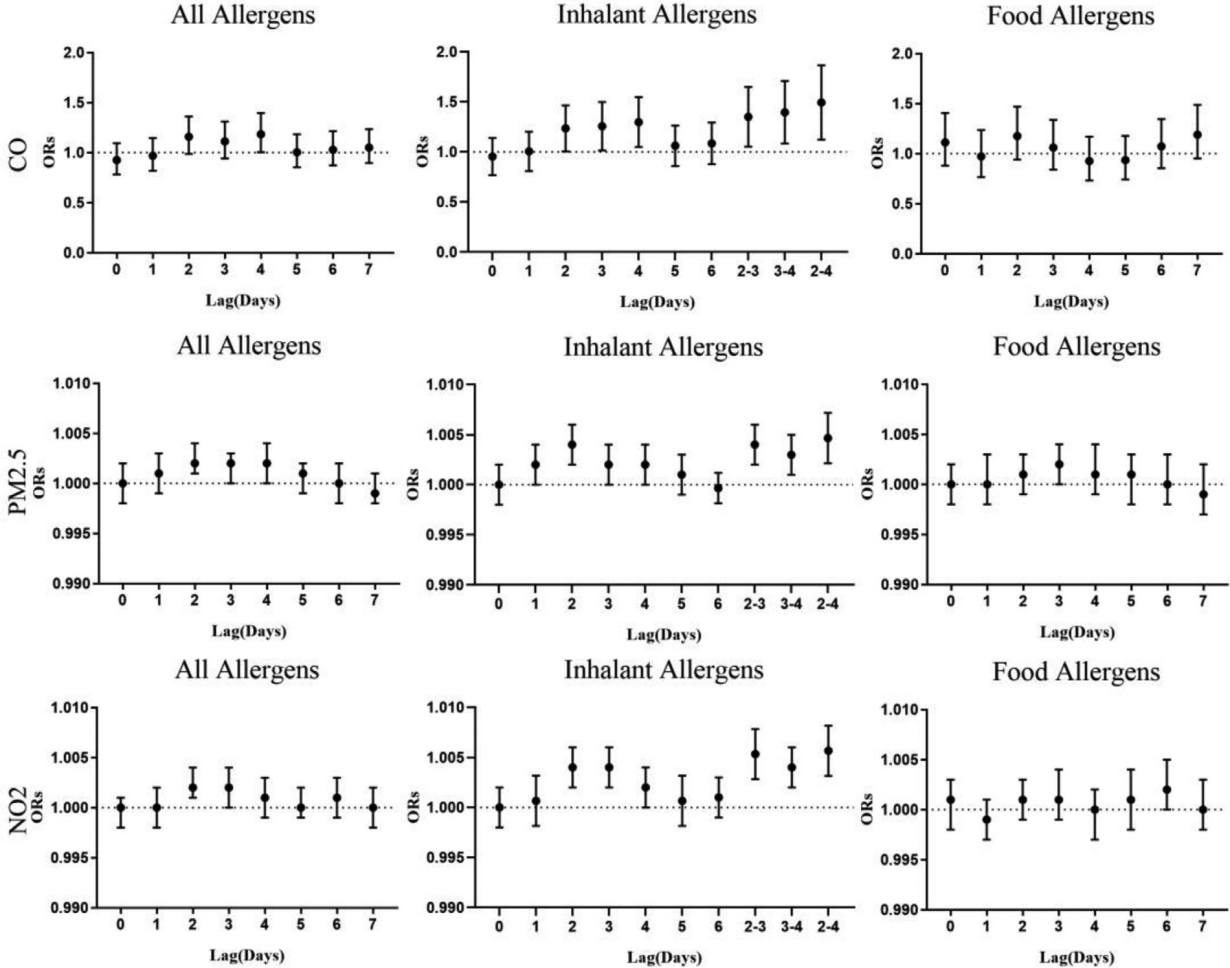
The association between short-term exposure to CO, PM_2.5_, NO_2_ and hospital visits for IgE-mediated allergy on different lag days in Guangzhou, China. Note: Error bars represent 95% confidence intervals of ORs. lag_0_, current day; lag_1_, previous 1 day; lag_2_, previous 2 days; lag_3_, previous 3 days; lag_4_, previous 4 days; lag_5_, previous 5 days; lag_6_, previous 6 days; lag_2–3_, 2-days moving average of lag_2_ and lag_3_; lag_3–4_, 2-days moving average of lag_3_ and lag_4_; lag_2–4_, 3-days moving average of lag_2_, lag_3_, and lag_4_; all allergens, allergic to any allergens; inhaled allergens, allergic to at least one of the d1, d2, e1, e5, w6, m3; food allergens, allergic to at least one of the f1, f2, f4, f13, f24, f14, f23.

The single-pollutant model of CO, PM_2.5_ and NO_2_ with various lag days was established to predict the risk of IgE-mediated allergy. Fig. 2 shows that compared with food allergens, these three air pollutants have significantly higher risk effects on inhaled allergy, particularly in lag_2–4_, and the corresponding ORs (95% CI) of CO, PM_2.5_ and NO_2_ are 1.464(1.139, 1.881), 1.005(1.002, 1.007) and 1.006(1.003, 1.008), respectively. Supplement Table 1, 2, and 3 reports that the exposures to CO, PM_2.5_ and NO_2_ (lag_2–4_) have the highest risks on allergic to house dust mite-specific molecules (*p* < 0.05). Besides, similar associations are also found between dermatophagoides farinae-specific molecules and PM_2.5_/NO_2_ concentrations (Supplement Table 2 and 3) while allergic to dog dander-specific molecules are observed to have a significant correlation with CO exposure in lag_3_ and lag_3–4_. (Supplement Table 1). Additionally, the parameters of the model selection criterion were additionally calculated to evaluate the performance of the candidate models. The results (Supplement Table 4) show that the air pollutant model of lag_2–4_ has the lowest AIC, −2 Log L and BIC.

**Fig. 3:**
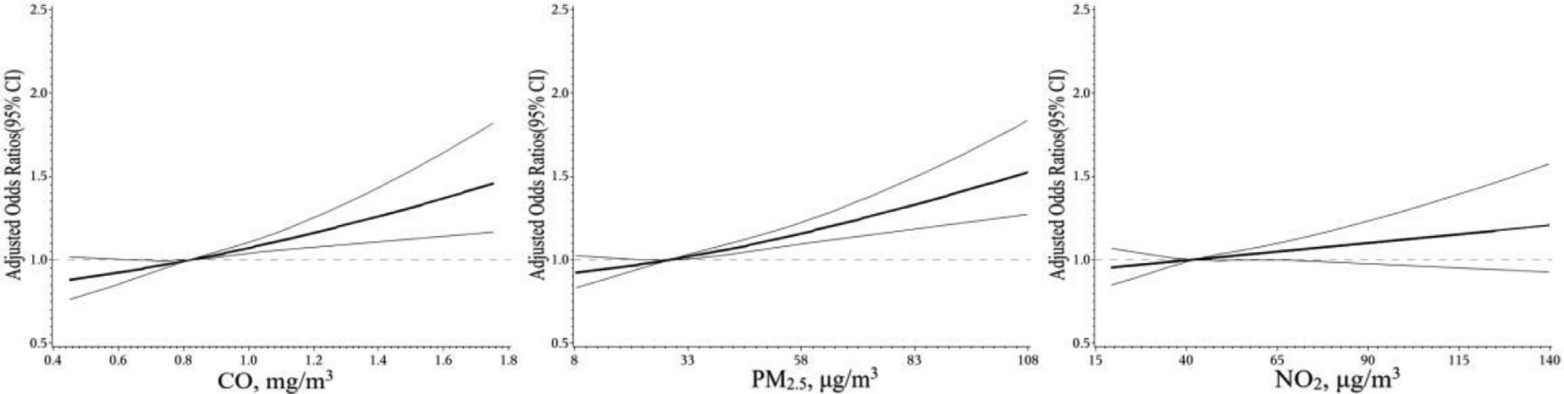
Exposure-response curves for the association between inhaled allergies and the 3-days (lag_2–4_) moving average of CO, PM_2.5_, and NO_2_ concentrations. Note: This figure shows the restrictive cubic spline curve for air pollutant concentrations with 95% confidence incidence. The ORs was adjusted with mean temperature, relative humidity and public holidays.

Fig. 3 depicts the exposure-response curve for the associations of the 3-day moving average (lag_2–4_) of CO, PM_2.5_, NO_2_ concentrations and allergic to specific inhaled allergens. After adjusting for potential confounding effects of relative humidity, mean temperature and public holidays, a positive linear association can be seen between the CO/PM_2.5_ exposure and the risk of inhaled allergies, and the associations become statistically significant when exposure levels are above the median (the reference value of ORs). However, no evidence supports that the NO_2_ levels can significantly enhance the risk of inhaled allergies. Supplement Figure 4, 5 and 6 are stratified analyses that mainly show the associations between CO, PM_2.5_ and NO_2_ levels (lag_2–4_) and sensitization to inhaled allergic molecules in this study. With the increase of CO concentration, the risks of allergic to house mite-specific molecules (d1) and dog dander-specific molecules (e5) as well as aspergillus fumigatus-specific molecules (m3) have an obvious rising trend. Moreover, compared with d1, the exposure-response curves seem to increase sharply when the CO levels go above the median at e5 and m3 (Supplement Figure 4). On the exposure to PM_2.5_ concentrations, the d1, d2 (dermatophagoides farinae-specific molecules), e5 and m3 specific allergic risks are all significantly increased (Supplement Figure 5). No similar associations are observed in NO_2_ (Supplement Figure 6).

Table 3 quantitatively demonstrates the short-term exposure effects of CO and PM_2.5_ concentration in two different ways (per SD or quartile increment). Five participants who were positive for inhaled allergens were excluded from the analysis due to failing to match their information about air pollutants for lag_2–4_. Supplement Table 5 shows that there are no significant differences in tIgE, gender and age between included and excluded patients (*p* > 0.05). Finally, a total of 28,739 cases (7456 blocks, 7456 cases vs. 21,283 controls) were included in the quantitative analysis of the associations of CO/PM_2.5_ (lag_2–4_) concentration and the risk of sensitization to specific inhaled allergens. In multi-pollutant models, the adjusted OR (95% CI) is increased by 8% (95% CI, 2%−15%) when CO levels per increment by 0.2 mg/m^3^, and is increased by 8% (95% CI, 2%−13%) just as PM_2.5_ levels per increment by 16.3 μg/m^3^. There is a significant correlation between the CO/PM_2.5_ levels and the risk of inhaled allergies (*p*_for_ _trend_ < 0.05). Compared with quartile 1, quartile 4 for CO and PM_2.5_ have the highest significant adjusted ORs (*p* < 0.05).

**Fig. 4:**
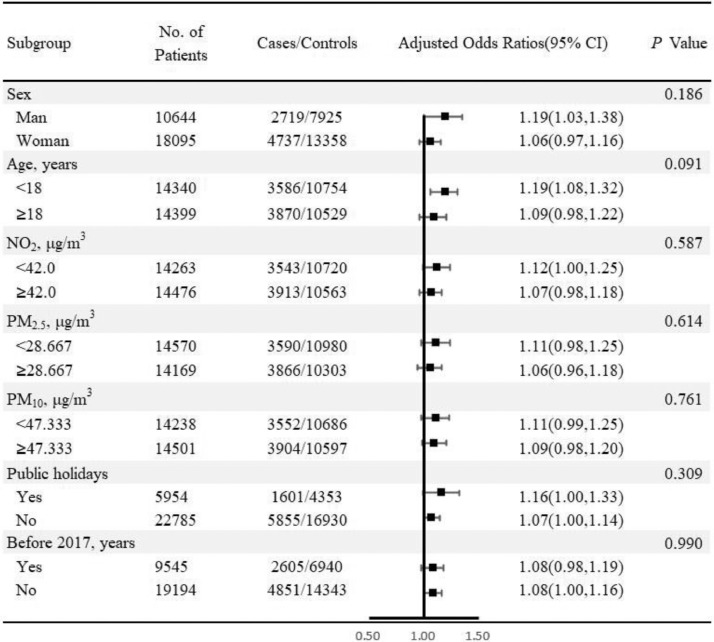
Subgroup analyses for the associations between carbon monoxide (per SD increment) and the risk of inhaled allergies. Note: The ORs was adjusted with mean temperature, relative humidity, SO_2_, and O_3__1 h.

**Fig. 5:**
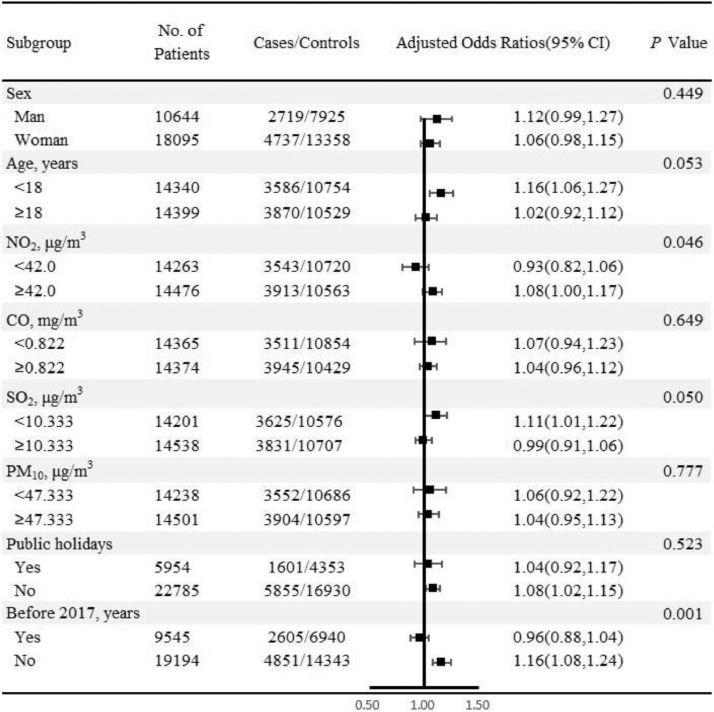
Subgroup analyses for the associations between particulate matter less than 2.5 μm (per SD increment) and the risk of inhaled allergies. Note: The ORs was adjusted with mean temperature, relative humidity, and O_3__1 h.

Fig. 4, Fig. 5 indicate the robustness of the correlation between CO/PM_2.5_ (lag_2–4_) levels (per SD increment) and the risk of inhaled allergies in various subgroups. A significantly positive association can be seen among youngsters under 18 years old [adjusted ORs (95% CI) are 1.19 (1.08, 1.32) in CO and 1.16 (1.06, 1.27) in PM_2.5_] but not in adults (≥18, years). Interestingly, NO_2_ is likely a potential modifier for the association between PM_2.5_ and the risk of inhaled allergies because the risk estimates for PM_2.5_ in higher NO_2_ (≥42.0, μg/m^3^) are statistically different from those in lower NO_2_ levels (*p* = 0.046). Similarly, the exposure risk of PM_2.5_ under a higher level of SO_2_ (≥10.333 μg/m^3^) is comparatively less than those of lower SO_2_ (<10.333 μg/m^3^) concentrations (*p* = 0.050). Furthermore, compared with the included patients before 2017, a significantly stronger positive association is observed between PM_2.5_ and the risk of inhaled allergies after 2017. No other factors serve as significant effect modifiers on the association between CO/PM_2.5_ exposure and the risk of inhaled allergies (*p* > 0.05).

## 4 Discussion

In this study, we reveal that the 3-day moving average (lag_2–4_) of CO, PM_2.5_ or NO_2_ is associated with the increased risk of hospital visits for inhaled allergies. In multi-pollutant models, the associations between CO/PM_2.5_ and the risk of inhaled allergies remain significant and are stronger in youngsters (<18, years). However, there is no significant difference by gender. Additionally, a significantly stronger association between PM_2.5_ exposure and hospital visits for inhaled allergy is observed in patients who are exposed to lower concentration of SO_2_ (<10.333 μg/m^3^) and higher levels of NO_2_ (≥42.0 μg/m^3^), as well as patients enrolled after 2017. Our findings are beneficial to strengthen the currently weak epidemiological evidence regarding the associations between acute air pollution and allergic sensitization.

To date, only a few epidemiological studies investigate the association between outdoor air pollution and atopic diseases or allergic sensitization, and most of them were conducted in high-income countries [18,28]. Consistent with our findings, a study from Germany [18] reported that the inhaled allergy is closely related to PM_2.5_ but not to NO_2_. However, a meta-analysis [29] suggested that no clear associations between air pollution exposure and the development of allergic sensitization in European birth cohorts. Besides, unlike previous studies, more robust correlations between CO level and inhaled allergies are found in our study, particularly in lag_2–4_ (ORs = 1.464). To our knowledge, we are the first to discover such an association. In contrast to prior studies, our study has the following outstanding features. (1) This is the first epidemiological study with a large sample size (*n* = 39,569) in southern China to investigate the association between daily hospital visits for IgE-mediated allergy and short-term exposure to air pollution, and the large sample size would yield higher statistical power. (2) Our study uses a case-crossover study design in which the case and several controls were originated in the same person. Therefore, inherent confounding factors can be addressed, and the results are more reliable. (3) The differences between subgroups can be observed using stratified analyses which would help us draw more reliable conclusions.

Many epidemiology studies demonstrated that short-term exposure to air pollutants is associated with the increased hospital admissions for respiratory disease. Consistent with other studies, we also demonstrate the effects of short-term exposure to air pollutants on the increased risk of IgE-mediated allergy. Additionally, we are the first to reveal that the 3-day moving average (lag_2–4_) of CO, PM_2.5_ or NO_2_ is associated with the increased risk of hospital visits for inhaled allergies. The potential biological mechanism is that the short-term exposure to air pollutants may have an independent effect on the risk of allergy and is likely to enhance the levels of allergy-characterized immunoglobulin (Ig) E. Besides, several experimental studies suggest that ambient air pollutants probably acting as the carrier is pertinent to the hypersensitivity mediated by mast cells and basophils. For instance, a study [30] performed in Mexico discloses that acute inhalation of PM_2.5_ acted as an adjuvant, like the aluminum hydroxide effect, triggering allergic asthma in a guinea pig model.

There is compelling evidence for the association between acute exposure to air pollution and hospital visits for cardiovascular, respiratory as well as gastrointestinal diseases [9,23,27], and the increased risk of oxidative damage and immune activation after exposure to air pollutants can explain such associations. Similarly, a transient increase in ambient air pollution might also increase the risk of allergic sensitization through induce systemic inflammation and oxidative stress. Additionally, the possibility of fine particulate matters (PM) enhancing allergic sensitization has been confirmed in mouse models [13], which suggests that once those air pollutants are inhaled and retained in the lung, they will enhance the inflammatory response and oxidative stress of the body. Yue et al. [11] demonstrate that maternal exposure of BALB/c mice to NO_2_ enhances the level of allergic asthma-characterized immunoglobulin in offspring, thus contributing to the increase of allergic symptoms. While no direct molecular mechanisms can interpret the associations between CO exposure and allergic responses based on current reviews, several studies have investigated the association between CO exposure and oxidative stress response of the body [31,32], and there exists evidence supporting the strong correlation between oxidative stress and allergic diseases [33], [34], [35]. Collectively, these findings indirectly demonstrate the biological mechanisms for the linkage between acute air pollution and allergic sensitization, and partially illustrate the lag effects of exposure to these air pollutants.

Interestingly, previous studies indicated that the association between PM_2.5_ levels and hospital admission for cardiovascular disease (CVD) was non-linear [27], while that between CO exposure and hospitalizations for CVD was linear [36]. However, based on our results, exposure-response associations between short-term air pollutants exposure (CO, PM_2.5_, NO_2_) and the risk of inhaled allergies are positively linear, and especially, the associations become statistically significant when the concentration of CO/PM_2.5_ gets higher (Fig. 3). The present study suggests that either per 0.2 mg/m^3^ increment of CO or per 16.3 μg/m^3^ increment of PM_2.5_ has an 8% increase in hospital visits for sensitization to specific inhaled allergens when other confounders are under control. We are the first to discover this quantitative relationship. Our finding would be beneficial to set air quality guidelines in the prevention of inhaled allergies in regions with similar social and demographic characteristics as Guangzhou. Additionally, prospective cohort studies are required to explore the relationship between air pollutants and food allergens because of limited evidence in this study.

The identification of more vulnerable subgroups that are exposed to air pollutions contributes to reducing the burden of allergic diseases using minimum public health-care resources. Our finding suggests that youngsters (<18, years) may be more sensitive to inhaled allergens than adults (≥18, years) under the short-term exposure to CO/PM_2.5_. Similar conclusions are also drawn by other studies [37], [38], [39], which declare that the occurrence of autoimmune or allergic disease is primarily affected by the lack of autoantibodies. Therefore, compared with adults, adolescents are more likely to suffer from autoimmune diseases and allergies. Nevertheless, unlike other studies [40] showing that women are much more sensitive to allergic diseases since the level of estrogens may increase the risk of allergic sensitization, our study reports that there is no evidence to confirm the difference between males and females. Accordingly, further evidence should be provided.

Previous similar studies do not adjust collinearity among correlated air pollutants in their multi-pollutant models. We believe that it is appropriate to utilize subgroup analyses to investigate the variation of risk between different levels of air pollution because it can avoid the risk of multi-collinearity while exploring the interaction between them. The results of the study show that the associations between PM_2.5_ concentration and inhaled allergies are likely to be modified by SO_2_ and NO_2_, we speculate that it is because of the influence of the size of particle matter in the process of allergy reaction [12]. SO_2_ and NO_2_ are recognized to share similar molecular mechanisms with PM_2.5_, which can result in systemic inflammation response [41,42].

Furthermore, we investigate the time variation of risk because of the obvious upgrade trend of daily admissions for acute allergic symptoms since 2017 (Supplement Figure 2). It can be observed that in patients enrolled after 2017, the risk of inhaled allergies on the exposure to PM_2.5_ is much higher than those enrolled before 2017. There are possible interpretations of this time variation as follows. Firstly, previous research reports that the average exposure level of PM_2.5_ in 74 key Chinese cities falls from 72.2 μg/m^3^ in 2013 to 47.0 μg/m^3^ in 2017 [43]. And the median level of PM_2.5_ exposure after 2017 (27.0 μg/m^3^) is significantly less than that before 2017 (38.0 μg/m^3^) in Guangzhou city. Therefore, the association between the short-term exposure to PM_2.5_ and the risk of sensitization to inhaled allergens is likely more robust in lower levels of ambient PM_2.5_. Secondly, the development of allergen test technology enables more allergic patients identified. Thirdly, in recent years, the development of traffic (partially due to the increasing popularity of cars within an ordinary family) increases the chance of exposure to PM_2.5_, thus increasing the risk of allergic diseases.

In this study, because of the variance of spending time between indoors and outdoors for participants, using the ambient concentration of air pollutants as exposure to correlate with IgE-mediated allergy can partly reduce the effect estimate of air pollutions [44]. Considering that the time of indoors and outdoors is different between a public holiday and working days, we used conditional logistic regression to estimate the association while adjusting for public holidays [27]. Moreover, subgroup analyses are also performed on exposure assessment whether the time was in public holidays, which provides more robust evidence to identify associations (Fig. 4 and 5). The results show that there is no significant difference between air pollutants and the risks of IgE-mediated allergy in a public holiday or not. Overall, although we cannot get an individual exposure time of indoors and outdoors, the statistics measures in this study can partly avoid the bias of using the ambient concentration of air pollutants as exposure.

Several limitations still require our attention. All patients are collected in the First Affiliated Hospital of Guangzhou Medical University, the enrolled participants only come from a single area might limit the generalizability of our findings to other populations who are exposed to different ambient air pollutant concentrations. Besides, as in similar studies [27,36], the individual exposure to air pollution, including indoor and outdoor exposure, is represented by 10 fixed-site monitors in Guangzhou city (https://www-app.gdeei.cn/eqpublish/index.html#/home/air-city/latest), this may lead to some measurement bias in our study. Nevertheless, the present study site is Guangzhou, one of the biggest cities in southern China that is equipped with a complete air monitoring instrument. Therefore, the measurement bias of air pollutants can partly be reduced in this situation.

In conclusion, we demonstrate that the short-term exposure to CO and PM_2.5_ is associated with increased hospital visits for the allergy caused by specific inhaled allergens in Guangzhou, southern China. We also reveal that either per 0.2 mg/m^3^ increment of CO or per 16.3 μg/m^3^ increment of PM_2.5_ has an 8% increase in hospital visits for sensitization to specific inhalant allergens on lag_2–4_ when other confounders are under control. Our findings should enable public health policymakers to recommend safety limitations on the exposure levels of outdoor air pollutants for those susceptible to inhaled allergies at the city-specific level which is similar to Guangzhou.

## Funding

This study was supported by the 10.13039/501100011322University of Macau (grant numbers: FHS-CRDA-029–002–2017 and MYRG2018–00, 071-FHS) as well as the 10.13039/501100003009Science and Technology Development Fund, Macau SAR (File no. 0004/2019/AFJ and 0011/2019/AKP). This work was also supported by the 10.13039/501100001809National Natural Science Foundation of China (81,871,736), the National Key Technology R&D Program (2018YFC1311902), the 10.13039/501100009330Guangdong Science and Technology Foundation (2019B030316028), the Guangzhou Municipal Health Foundation (20191A011073), and the Guangzhou Science and Technology Foundation (201,804,020,043).

## Contributors

Xiangqing Hou, Huimin Huang, Baoqing Sun and Xiaohua Douglas Zhang designed the study. Xiangqing Hou, Baoqing Sun and Xiaohua Douglas Zhang accessed the raw data. Huimin Huang, Haisheng Hu, Dandan Wang completed the data management and data cleaning as well as interpretation. Xiangqing Hou and Xiaohua Douglas Zhang performed the statistical analysis. Xiangqing Hou and Huimin Huang drafted the manuscript. Xiangqing Hou, Baoqing Sun and Xiaohua Douglas Zhang edited the manuscript. All authors contributed to critically revision of the manuscript, and approved the final version. Xiangqing Hou, Baoqing Sun and Xiaohua Douglas Zhang had full access to the raw data in this study and had final responsibility for the decision to submit it for publication.

## Availability of data and materials

The datasets used and/or analysed during the current study are available from the corresponding author on reasonable request.

## Declaration of Competing Interest

All authors declare no potential conflicts of interest.

## Appendix Supplementary materials

Image, application 1
