# Supplementary material for: Short-term exposure to ambient air pollution and hospital visits for IgE-mediated allergy: A time-stratified case-crossover study in southern China from 2012 to 2019
Source: eClinicalMedicine. 2021 Jun 10;37:100949. doi: 10.1016/j.eclinm.2021.100949 (PMC8343265; doi:10.1016/j.eclinm.2021.100949)
Supplement: Supplementary file 1 [file mmc1.docx]

**Supplement Figure 1.**

**
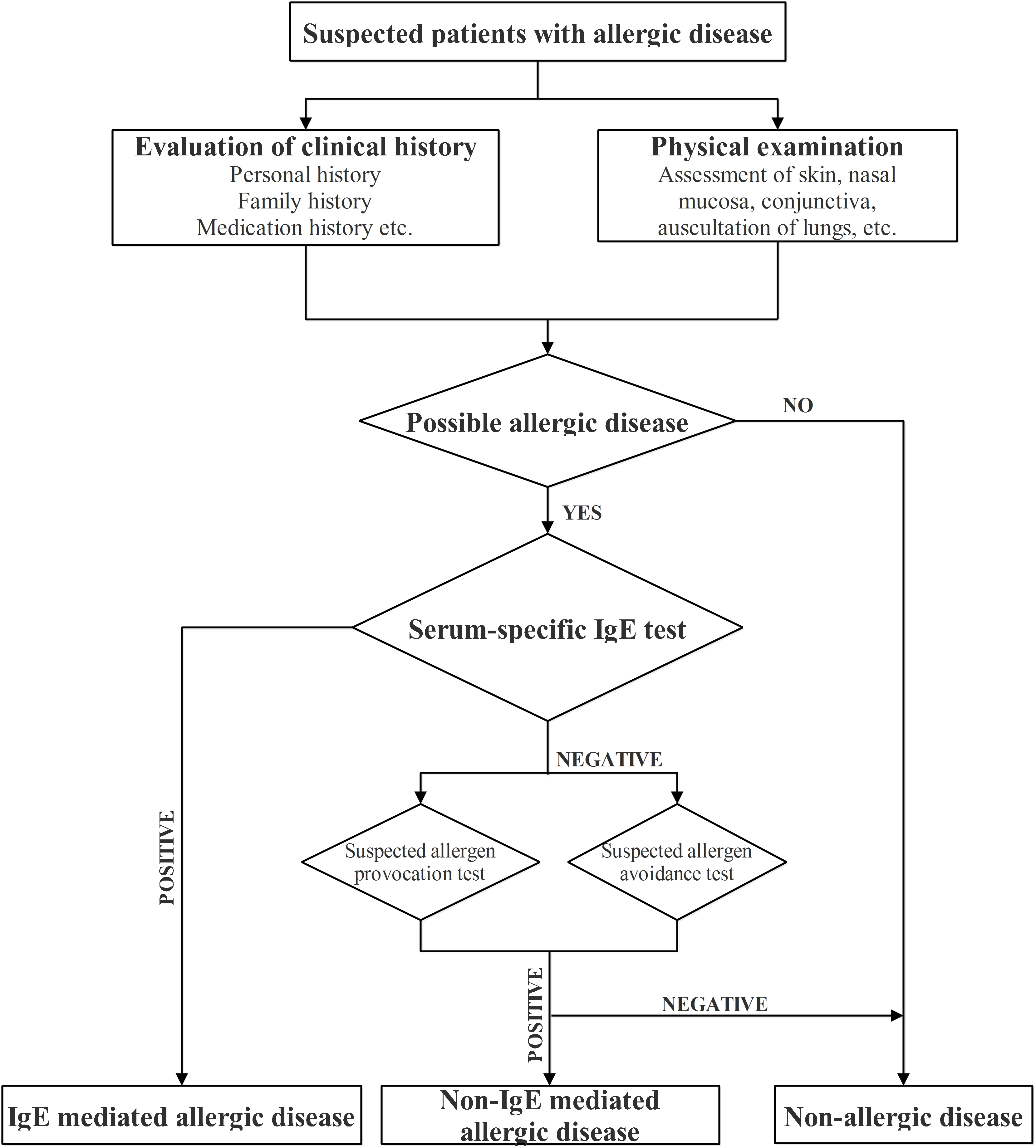
**

**Supplement Figure 2.**

**
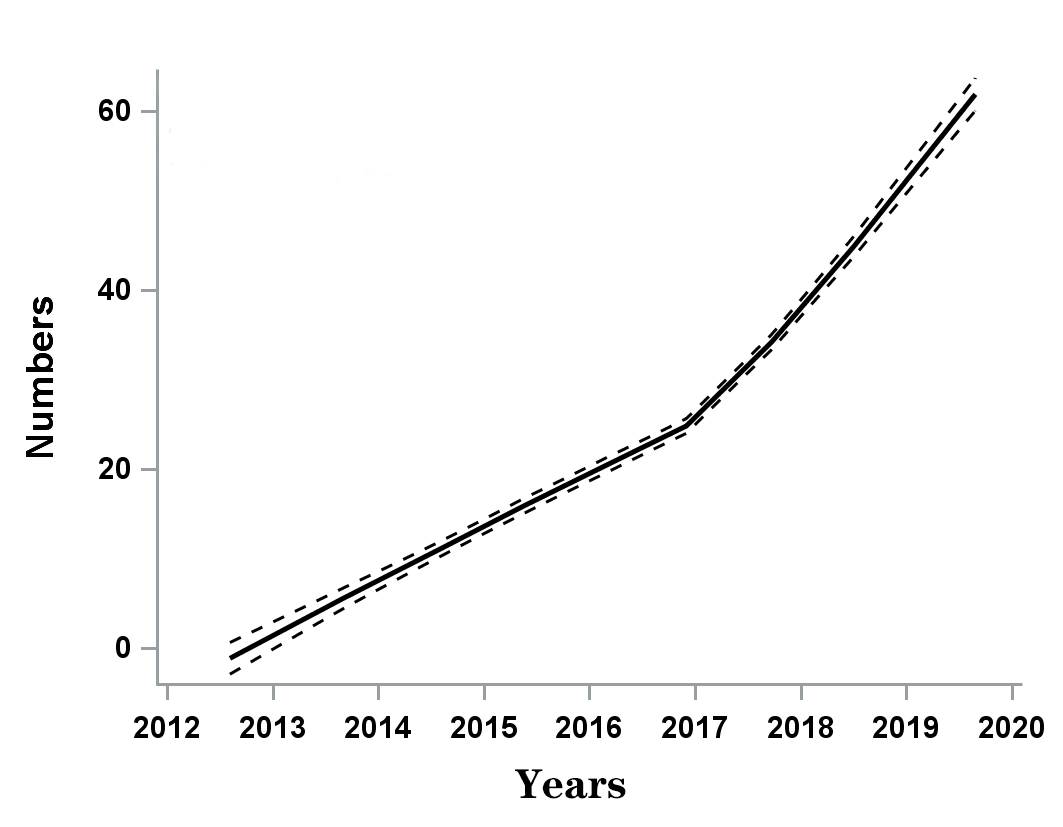
**

**Supplement Figure 3.**


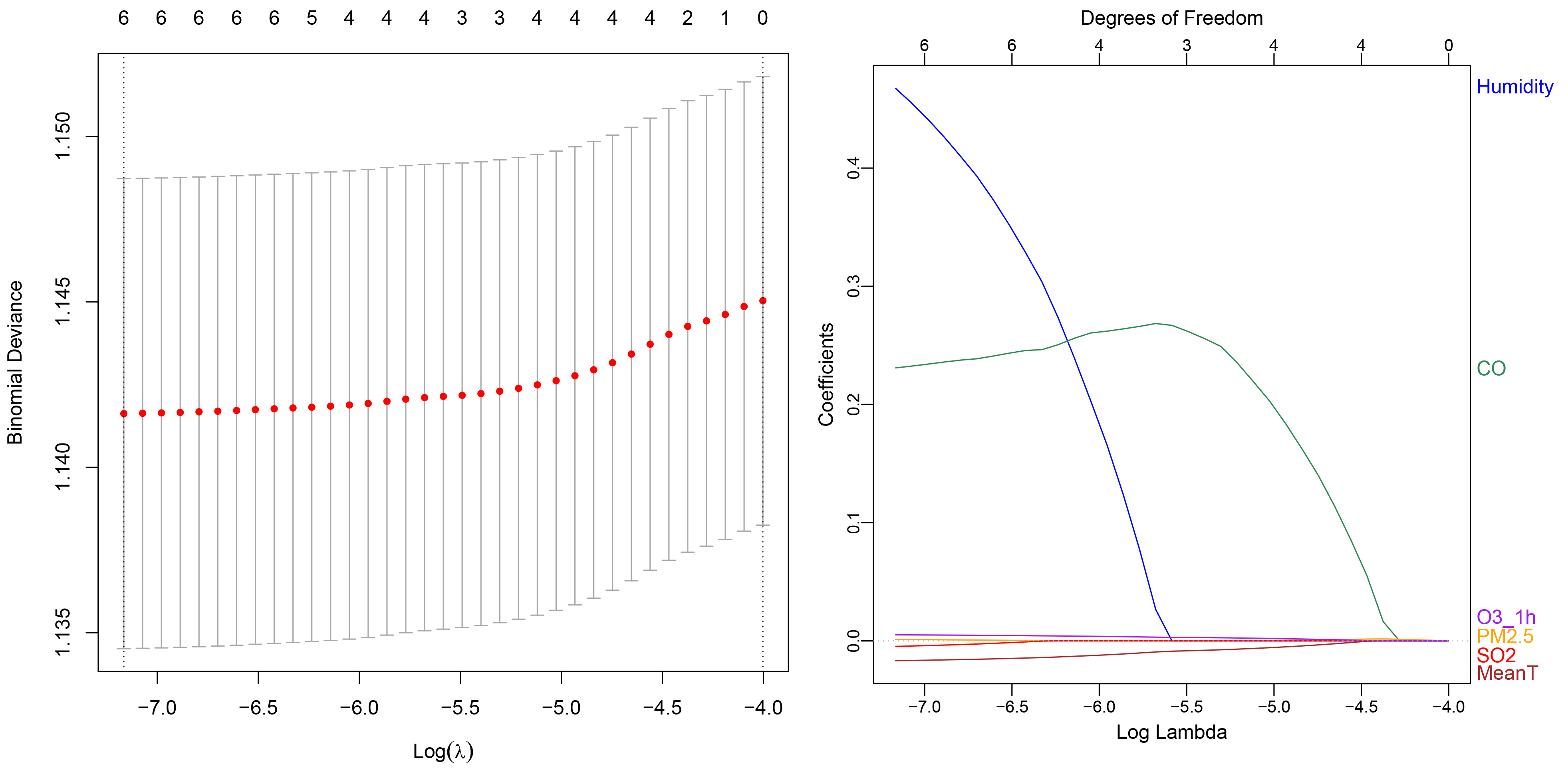


**Supplement Figure 4.**

**
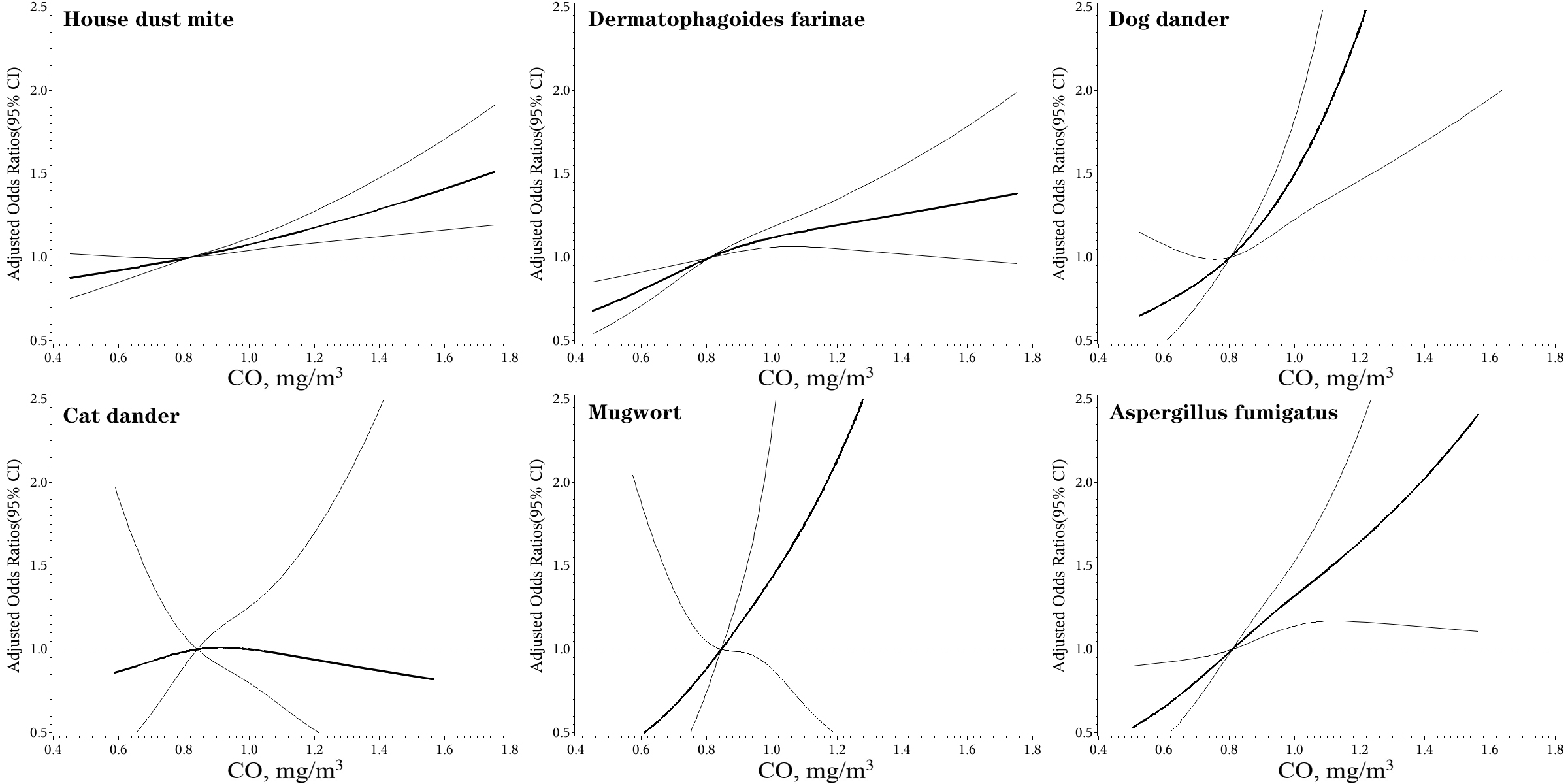
**

**Supplement Figure 5.**

**
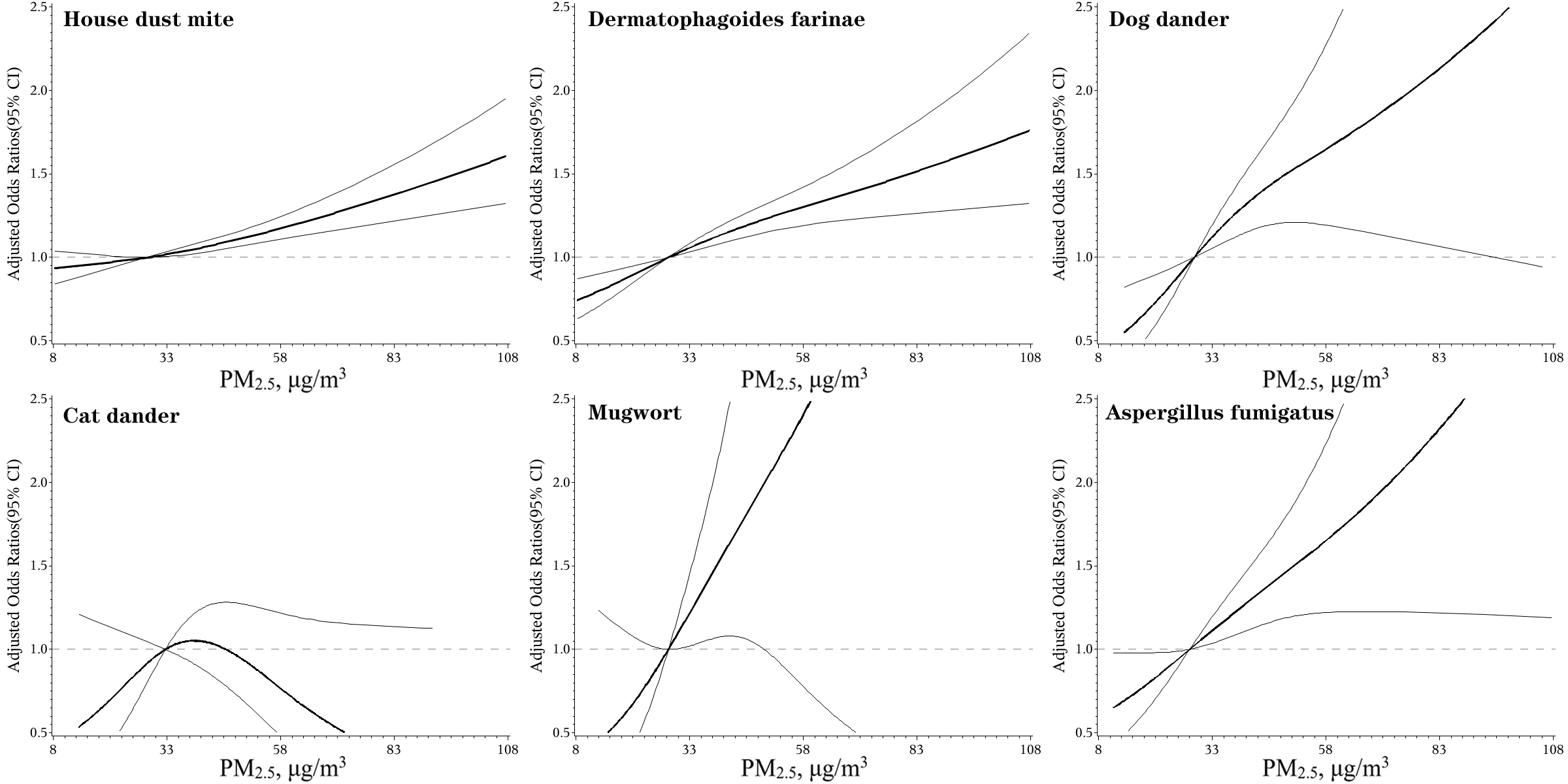
**

**Supplement Figure 6.**

**
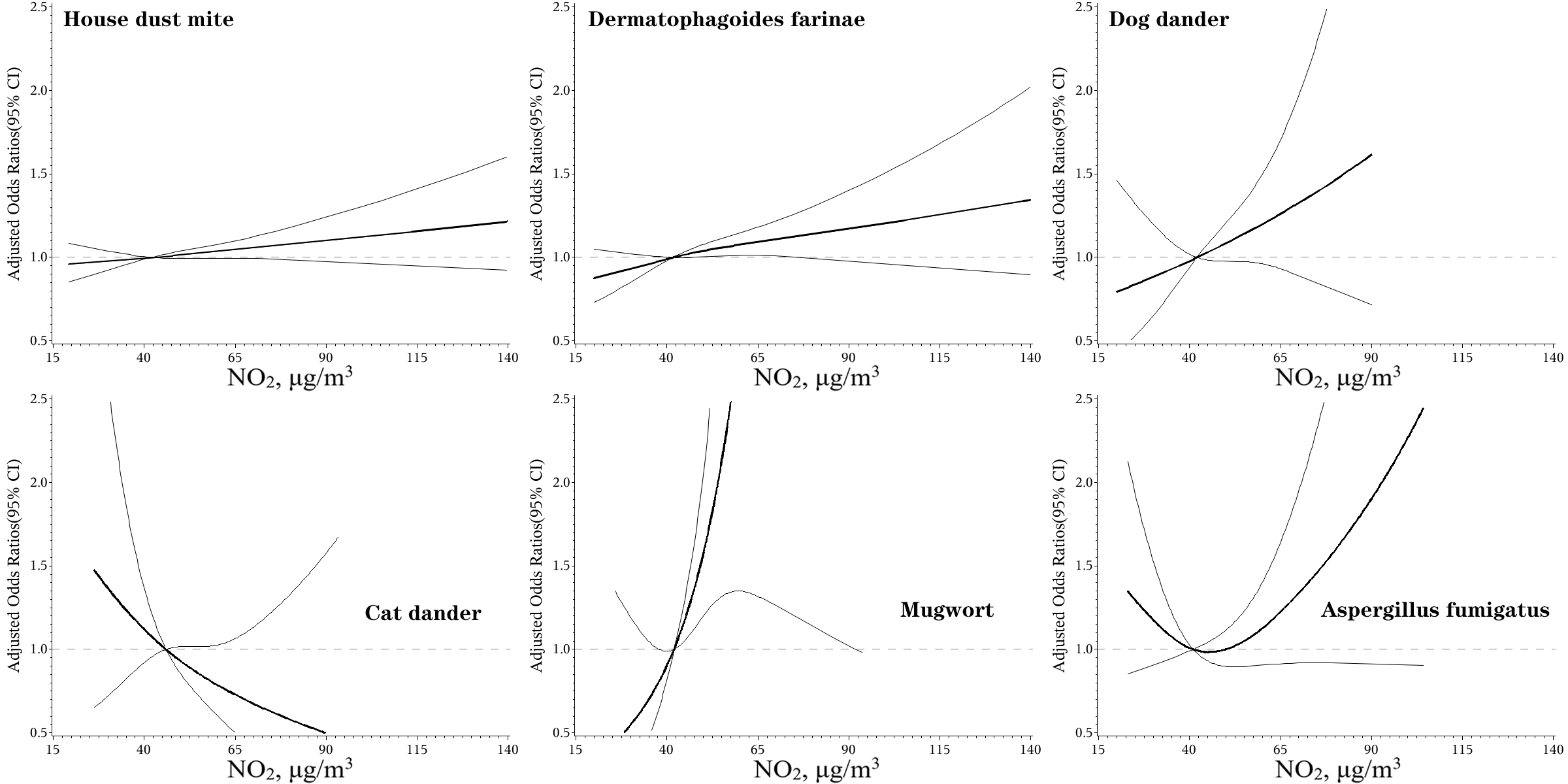
**

**Supplement Figure 1.** Diagnostic flowchart of allergic diseases.

**Supplement Figure 2.** Daily hospital visits patients with acute allergic symptoms in the First Affiliated Hospital of Guangzhou Medical University from October 2012 to September 2019.

**Note:** This figure shows the loess curve for changes in the number of participants with 95% confidence incidence.

**Supplement Figure 3.** LASSO coefficient profiles of 6 selected variable in the 10-fold cross validation.

**Note:** CO, carbon monoxide; SO2, sulfur dioxide; O3_1h, daily maximum 1-hour average ozone concentrations; PM2.5, particulate matter less than 2.5 μm; MeanT, mean temperature.

**Supplement Figure 4.** The association between the 3-days moving average (lag_2-4_) of carbon monoxide (CO) concentrations and the risk of atopic diseases related to specific inhalant allergens.

**Note:** This figure shows the cubic spline curve for CO concentrations with 95% confidence incidence. The ORs was adjusted with mean temperature, relative humidity as well as public holidays.

**Supplement Figure 5.** The association between the 3-days moving average (lag_2-4_) of particulate matter less than 2.5 μm (PM_2.5_) concentrations and the risk of atopic diseases related to specific inhalant allergens.

**Note:** This figure shows the cubic spline curve for PM_2.5_ concentrations with 95% confidence incidence. The ORs was adjusted with mean temperature, relative humidity as well as public holidays.

**Supplement Figure 6.** The association between the 3-days moving average (lag_2-4_) of nitrogen dioxide (NO_2_) concentrations and the risk of atopic diseases related to specific inhalant allergens.

**Note:** This figure shows the cubic spline curve for NO_2_ concentrations with 95% confidence incidence. The ORs was adjusted with mean temperature, relative humidity as well as public holidays.

**Abbreviations:** tIgE, total IgE; d1, house dust mite; d2, dermatophagoides farinae; m3, aspergillus fumigatus; e1, cat dander; e5, dog dander; w6, mugwort; f1, egg white; f2, milk; f4, wheat; f13, peanut; f24, shrimp; f14, soy; f23, crab; CO, carbon monoxide; NO_2_, nitrogen dioxide; SO_2_, sulfur dioxide; O_3__1h, daily maximum 1-hour average ozone concentrations; PM_2.5_ and PM_10_, particulate matter less than 2.5 μm and 10 μm in aerodynamic diameter; AR, allergic rhinitis; AD, atopic dermatitis; SD, standard deviation; CI, confidence interval; ORs, odds ratios; Q_1_, the first quartile; Q_2_, the second quartile; Q_3_, the third quartile; Q_4_, the fourth quartile; CI, confidence interval; AIC, Akaike Information Criterion; BIC, Bayesian Information Criterion.

**Supplement Table1.** Odds ratios (95% CI) for specific inhaled IgE-mediated allergy per unit increment in CO concentrations on different lag days.

| Allergens | Lag days | | | | | | | | | |
| --- | --- | --- | --- | --- | --- | --- | --- | --- | --- | --- |
|  | 0 | 1 | 2 | 3 | 4 | 5 | 6 | 2-3 | 3-4 | 2-4 |
| d1 | 0.973  (0.792-1.195) | 0.984  (0.801-1.208) | 1.212  (0.997-1.473) | 1.206  (0.986- 1.477) | 1.297*  (1.060-1.586) | 0.992  (0.812-1.212) | 1.017  (0.831-1.245) | 1.299*  (1.029-1.640) | 1.359*  (1.074-1.719) | 1.447*  (1.113-1.880) |
| d2 | 0.976  (0.714-1.333) | 0.939  (0.689-1.280) | 1.270  (0.951-1.694) | 1.080  (0.789-1.478) | 0.998  (0.734-1.358) | 1.008  (0.742-1.368) | 0.769  (0.561-1.055) | 1.262  (0.882-1.807) | 1.054  (0.727-1.528) | 1.230  (0.812-1.861) |
| m3 | 0.891  (0.388-2.044) | 1.167  (0.493-2.766) | 1.262  (0.529-3.011) | 0.821  (0.373-1.808) | 1.141  (0.518-2.514) | 0.738  (0.313-1.737) | 0.769  (0.330-1.789) | 0.994  (0.345-2.863) | 0.948  (0.346-2.596) | 1.106  (0.325-3.763) |
| e5 | 0.953  (0.354-2.567) | 1.066  (0.411-2.763) | 1.028  (0.431-2.450) | 2.444*  (1.089-5.485) | 1.656  (0.731-3.751) | 1.698  (0.679-4.248) | 0.849  (0.341-2.112) | 2.163  (0.762-6.145) | 2.739*  (1.034-7.259) | 2.883  (0.859-9.680) |
| e1 | 1.216  (0.285-5.184) | 0.541  (0.079-3.681) | 0.771  (0.191-3.120) | 1.046  (0.238-4.591) | 1.404  (0.385-5.122) | 1.474  (0.438-4.960) | 0.999  (0.303-3.299) | 0.849  (0.154-4.671) | 1.361  (0.258-7.185) | 1.113  (0.169-7.341) |
| w6 | 0.645  (0.096-4.331) | 0.680  (0.076-6.079) | 1.382  (0.162-11.798) | 0.766  (0.062-9.478) | 1.426  (0.241-8.446) | 1.399  (0.205-9.557) | 1.496  (0.148-15.156) | 1.135  (0.056-23.123) | 1.273  (0.092-17.619) | 1.630  (0.065-41.090) |

**Note:** d1, house dust mite; d2, dermatophagoides farinae; m3, aspergillus fumigatus; e1, cat dander; e5, dog dander; w6, mugwort; lag_0_, current day; lag_1_, previous 1 day; lag_2_, previous 2 days; lag_3_, previous 3 days; lag_4_, previous 4 days; lag_5_, previous 5 days; lag_6_, previous 6 days; lag_2-3_, 2-days moving average of lag_2_ and lag_3_; lag_3-4_, 2-days moving average of lag_3_ and lag_4_; lag_2-4_, 3-days moving average of lag_2_, lag_3_, and lag_4_; 95%CI, 95% Confidence Interval; **P*<0.05.

**Supplement Table2.** Odds ratios (95% CI) for specific inhaled IgE-mediated allergy per unit increment in PM_2.5_ concentrations on different lag days.

| Allergens | Lag days | | | | | | | | | |
| --- | --- | --- | --- | --- | --- | --- | --- | --- | --- | --- |
|  | 0 | 1 | 2 | 3 | 4 | 5 | 6 | 2-3 | 3-4 | 2-4 |
| d1 | 1.000  (0.998-1.002) | 1.002  (1.000-1.004) | 1.004¶  (1.002-1.006) | 1.002*  (1.000-1.004) | 1.003*  (1.000-1.005) | 1.000  (0.998-1.002) | 0.999  (0.997-1.002) | 1.004¶  (1.002-1.006) | 1.003*  (1.001-1.005) | 1.005¶  (1.002-1.007) |
| d2 | 1.002  (0.999-1.006) | 1.002  (0.999-1.005) | 1.005*  (1.001-1.008) | 1.004*  (1.001-1.007) | 1.002  (0.999-1.005) | 1.001  (0.998-1.004) | 0.998  (0.995-1.001) | 1.006*  (1.002-1.009) | 1.004*  (1.000-1.007) | 1.006*  (1.002-1.010) |
| m3 | 1.000  (0.991-1.009) | 1.002  (0.994-1.010) | 1.001  (0.993-1.010) | 0.999  (0.991-1.008) | 1.003  (0.994-1.011) | 0.999  (0.989-1.009) | 0.997  (0.989-1.006) | 1.000  (0.990-1.011) | 1.002  (0.990-1.013) | 1.003  (0.989-1.016) |
| e5 | 1.003  (0.994-1.013) | 1.005  (0.995-1.015) | 1.001  (0.990-1.012) | 1.003  (0.993-1.013) | 1.005  (0.996-1.014) | 1.005  (0.996-1.014) | 0.999  (0.988-1.010) | 1.003  (0.990-1.016) | 1.007  (0.995-1.019) | 1.008  (0.993-1.023) |
| e1 | 1.007  (0.993-1.021) | 1.001  (0.984-1.018) | 0.995  (0.978-1.012) | 0.995  (0.980-1.010) | 1.004  (0.990-1.018) | 1.003  (0.987-1.020) | 1.001  (0.988-1.013) | 0.990  (0.968-1.013) | 0.999  (0.979-1.020) | 0.995  (0.968-1.023) |
| w6 | 0.998  (0.976-1.019) | 0.990  (0.968-1.013) | 1.006  (0.982-1.030) | 0.997  (0.962-1.033) | 1.004  (0.982-1.027) | 1.001  (0.979-1.025) | 1.008  (0.983-1.034) | 1.005  (0.970-1.040) | 1.004  (0.967-1.043) | 1.009  (0.969-1.050) |

**Note:** d1, house dust mite; d2, dermatophagoides farinae; m3, aspergillus fumigatus; e1, cat dander; e5, dog dander; w6, mugwort; lag_0_, current day; lag_1_, previous 1 day; lag_2_, previous 2 days; lag_3_, previous 3 days; lag_4_, previous 4 days; lag_5_, previous 5 days; lag_6_, previous 6 days; lag_2-3_, 2-days moving average of lag_2_ and lag_3_; lag_3-4_, 2-days moving average of lag_3_ and lag_4_; lag_2-4_, 3-days moving average of lag_2_, lag_3_, and lag_4_; 95%CI, 95% Confidence Interval; **P*<0.05; ¶*P*≤0.001.

**Supplement Table3.** Odds ratios (95% CI) for specific inhaled IgE-mediated allergy per unit increment in NO_2_ concentrations on different lag days.

| Allergens | Lag days | | | | | | | | | |
| --- | --- | --- | --- | --- | --- | --- | --- | --- | --- | --- |
|  | 0 | 1 | 2 | 3 | 4 | 5 | 6 | 2-3 | 3-4 | 2-4 |
| d1 | 1.000  (0.998-1.003) | 1.000  (0.998-1.003) | 1.004¶  (1.002-1.006) | 1.004¶  (1.001-1.006) | 1.002  (1.000-1.004) | 1.000  (0.998-1.002) | 1.000  (0.998-1.002) | 1.005¶  (1.003-1.008) | 1.004*  (1.001-1.006) | 1.005¶  (1.003-1.008) |
| d2 | 1.002  (0.998-1.005) | 1.003  (0.999-1.006) | 1.005*  (1.002-1.008) | 1.005*  (1.001-1.008) | 1.003  (1.000-1.007) | 1.002  (0.998-1.006) | 0.997  (0.993-1.001) | 1.007¶  (1.003-1.010) | 1.005*  (1.001-1.009) | 1.008¶  (1.003-1.012) |
| m3 | 1.000  (0.990-1.009) | 1.000  (0.991-1.009) | 1.003  (0.994-1.013) | 1.000  (0.990-1.010) | 1.000  (0.990-1.009) | 0.995  (0.984-1.006) | 0.997  (0.987-1.008) | 1.003  (0.990-1.017) | 1.000  (0.986-1.013) | 1.003  (0.986-1.020) |
| e5 | 1.006  (0.994-1.018) | 1.007  (0.996-1.019) | 1.000  (0.989-1.011) | 1.003  (0.992-1.014) | 1.003  (0.993-1.013) | 1.007  (0.997-1.017) | 1.001  (0.990-1.013) | 1.002  (0.998-1.017) | 1.005  (0.991-1.018) | 1.005  (0.988-1.022) |
| e1 | 1.010  (0.991-1.030) | 0.999  (0.982-1.017) | 0.999  (0.982-1.016) | 0.994  (0.977-1.012) | 1.010  (0.993-1.028) | 1.001  (0.984-1.019) | 0.998  (0.983-1.014) | 0.994  (0.971-1.018) | 1.005  (0.980-1.030) | 1.004  (0.973-1.037) |
| w6 | 0.991  (0.970-1.013) | 0.992  (0.966-1.019) | 1.006  (0.983-1.029) | 0.999  (0.957-1.042) | 1.003  (0.976-1.031) | 1.006  (0.986-1.026) | 1.008  (0.985-1.030) | 1.008  (0.969-1.048) | 1.003  (0.960-1.048) | 1.012  (0.963-1.063) |

**Note:** d1, house dust mite; d2, dermatophagoides farinae; m3, aspergillus fumigatus; e1, cat dander; e5, dog dander; w6, mugwort; lag_0_, current day; lag_1_, previous 1 day; lag_2_, previous 2 days; lag_3_, previous 3 days; lag_4_, previous 4 days; lag_5_, previous 5 days; lag_6_, previous 6 days; lag_2-3_, 2-days moving average of lag_2_ and lag_3_; lag_3-4_, 2-days moving average of lag_3_ and lag_4_; lag_2-4_, 3-days moving average of lag_2_, lag_3_, and lag_4_; 95%CI, 95% Confidence Interval; **P*<0.05; ¶*P*≤0.001.

**Supplement Table4.** Model fitting parameter of various lag days

| Parameter | Lag_2_ | Lag_3_ | Lag_4_ | Lag_2-3_ | Lag_3-4_ | Lag_2-4_ |
| --- | --- | --- | --- | --- | --- | --- |
| Variables |  |  |  |  |  |  |
| *CO* |  |  |  |  |  |  |
| AIC | 27916.05 | 27910.65 | 27904.15 | 27909.18 | 27902.91 | 27901.63 |
| -2 Log L | 27914.05 | 27908.65 | 27902.15 | 27907.18 | 27900.91 | 27899.63 |
| BIC | 27924.32 | 27918.92 | 27912.42 | 27917.45 | 27911.17 | 27909.90 |
| *PM_2.5_* |  |  |  |  |  |  |
| AIC | 27904.60 | 27909.66 | 27905.26 | 27902.15 | 27903.41 | 27897.08 |
| -2 Log L | 27902.60 | 27907.66 | 27903.26 | 27900.15 | 27901.41 | 27895.08 |
| BIC | 27912.87 | 27917.93 | 27913.52 | 27910.41 | 27911.67 | 27905.34 |
| *NO_2_* |  |  |  |  |  |  |
| AIC | 27904.38 | 27901.25 | 27906.24 | 27895.65 | 27899.33 | 27892.89 |
| -2 Log L | 27902.38 | 27899.25 | 27904.24 | 27893.65 | 27897.33 | 27890.89 |
| BIC | 27912.64 | 27909.51 | 27914.51 | 27903.91 | 27907.59 | 27901.16 |

**Note:** AIC, Akaike Information Criterion; BIC, Bayesian Information Criterion.

**Supplement Table5.** The comparison of descriptive characteristics between included and excluded participants

| **Variables** | **Included participants**  **N=34549** | **Excluded participants**  **N=420** | ***P* value** |
| --- | --- | --- | --- |
| tIgE, kU/L | 92.4(31.3,265.0) | 80.5(30.1,247.5) | 0.097 |
| Gender, % |  |  | 0.896 |
| Man | 14670(42.5) | 177(42.1) |  |
| Woman | 19879(57.5) | 243(57.9) |  |
| Age, year |  |  | 0.879 |
| ＜18 | 16816(48.7) | 206(49.0) |  |
| ≥18 | 17733(51.3) | 214(51.0) |  |

**Note:** The excluded participants including failing to match information about relative humidity, temperature or air pollutions with current day (n=415) as well as with lag_2-4_ (n=5); tIgE: total IgE.
